# The Diagnosis of Pregnancy

**Published:** 1906-11-03

**Authors:** F. C. McCann

**Affiliations:** Physician to the Samaritan Free Hospital for Women; Lecturer on Gynæcology, Medical Graduates' College and Polyclinic


### THE DIAGNOSIS OF PREGNANCY.

By Dr. F. C. McCann, M.D.(Edin.), Physician to the Samaritan Free Hospital for Women; Lecturer on Gynæcology, Medical Graduates' College and Polyclinic.

(Précis of a Lecture at the Polyclinic College.)

This subject is one of the greatest importance in practice, as error in connection with it may be disastrous both to the practitioner and the patient. Its difficulties vary with the earlier and the later stages of pregnancy. In the later months the diagnosis is arrived at through discovering the presence of the fœtus *in utero*. In the earlier months the changes produced on the genital organs as a result of conception are important, and a thorough and systematic examination of each case is necessary. Begin with the breasts, pass to the abdomen, and then make a thorough bimanual examination.

Most mistakes in diagnosing pregnancy are made through lack of system and careless examination. The breast signs are important in first pregnancies, but may be entirely absent in subsequent pregnancies, or may be so slight as to be unrecognisable. One makes out enlargement, hardening, development of superficial veins, pigmentation round the nipple, the develoment of the secondary areola, and Montgomery's tubercles, which are, like the breast itself, modified sebaceous glands. In conducting an abdominal examination, first inspect the vulva and note the presence or absence of blue discoloration. It is most marked round the urethral orifice and just at the entrance of the vagina. It may be entirely absent in the first pregnancy, and it may be present, as a result of chronic venous engorgement, in non-pregnant women who have had children, and it may be associated with large fibroid or ovarian tumour, and quite exceptionally with hepatic pulmonary or cardiac disease. Note the increase in softness of the vaginal walls and the increase of moisture there and at the cervix.

The vaginal portion of the cervix is described as being softened during pregnancy—one of the points which is remembered from student days. Other conditions in which softening is found are the presence of fibroids, pelvic inflammation, chronic metritis, and slight uterine prolapse; so that this alone is not a sure sign. The cervix may be examined through a speculum. If the mucous membrane has a well-marked reddish-blue appearance it affords a valuable indication. Examination of the uterus itself affords the most important aid in the diagnosis of pregnancy, especially the estimation of its consistence and size. The uterus enlarges in consequence of the growth of the impregnated ovum. It enlarges first in its transverse diameter owing to the thickening of the lateral walls. Later, as the ovum increases in growth, it separates the anterior from the posterior wall, giving an antero-posterior enlargement, resulting in a globular outline apparent at about 2½ to 3 months. The consistence of the uterus is a part of gynæcological diagnosis not sufficiently insisted on. The fingers should be educated thoroughly to appreciate the varieties: (1) normal consistency of the unimpregnated uterus; (2) that associated with pregnancy; (3) the impression associated with chronic metritis or with myoma. The normal uterus is firm and difficult to indent. During pregnancy it becomes doughy, and in chronic metritis it is so firm as to make indentation practically impossible; hence the term fibrotic uterus has been employed. This altered consistency affects both the body and the cervix. The fact that the ovum is growing in the upper part of the uterus, and the cervix is not so much softened as the lower uterine segment, gives rise on examination to Hegar's sign, which, if practised and once appreciated, will prove helpful in the diagnosis of early pregnancy. This sign is elicited bimanually by pressing downwards the external hand and pressing upwards with the internal fingers. They will almost meet at the level of the lower uterine segment. This is important in cases of dead fœtus or where products of miscarriage have been retained. In the later stages of pregnancy the problem is rather easier. Abdominal examination should reveal the presence of the fœtal parts, the fœtal movements (from the fourth month), or the sounds of the fœtal heart (from the commencement of the sixth month), and also the uterine souffle.

Certain cases illustrate the difficulties and the common mistakes of diagnosis of pregnancy. It is difficult in the early months to distinguish pregnancy from chronic metritis. In chronic metritis Hegar's sign *cannot* be made out; there is not the doughy feeling, and instead of having amenorrhœa, the presence of irregular hæmorrhages, or regular menstruation with tenderness on examination, may help the diagnosis. A dead or dying fœtus gives more trouble than probably any other pelvic condition, for in such cases the patient expects to be told if she is pregnant, and, if so, whether she shall miscarry. Hegar's sign helps here, for if it is present the patient is pregnant. Certain cases illustrate the notorious fallaciousness of histories. The physical examination must alone be relied on, and not what the patient relates.

Mistakes made in the early months may arise from well-marked anteflexion of the pregnant uterus. In these cases it is useful to press up the fundus uteri so as to undo the flexion and elicit Hegar's sign. Examination per rectum enables a much larger uterine area to be palpated than is possible through the vagina. Irregular growth of the pregnant uterus makes the diagnosis difficult in the early months. If the patient is troublesome to examine recommend an anæsthetic. These cases are puzzling because they simulate a cyst of the broad ligament. If there is doubt examine per rectum or under an anæsthetic. In some cases where the wall of the uterus is thin and the amount of the liquor amnii excessive there is an exact simulation of the conditions found in an ovarian cyst. In such cases (1) rhythmic contractions of the pregnant uterus can be felt if the hand is placed on the abdomen. Lawson Tait used to say that this was the most certain sign of pregnancy; (2) ballottement. If these two indications are met with the uterus is pregnant. The fœtal sounds may not be heard if a monstrosity is being dealt with. Spurious pregnancy—pseudocyesis—is a common cause of mistaken diagnosis in unmarried people, in those recently married, and in sterile women both before and after the menopause. Examination reveals a hard elastic uterus not enlarged. In doubt recommend an anæsthetic. The phantom tumour has not been satisfactorily explained. It may have to do with the costal respiration and fixation of the diaphragm. Patients approaching the menopause sometimes present difficulties in diagnosis. In one case a patient who had been married ten years and near the menopause was suspected of having a tumour. This subsequently proved to be a pregnancy. Another patient was sent as a case of tumour where the trouble was due to increased deposit of omental fat. Fibroid tumour is another condition apt to be mistaken for pregnancy. When large, such tumours should not give much trouble. As a rule the patient has increased menstrual loss, the tumour is firm, no rhythmical contractions of the uterus, but there may be a well-marked uterine souffle—no fœtal heart-sounds or changes in the vulva, no increased softness of the cervix. It is difficult to differentiate this from the presence of a dead fœtus. It may also give rise to an imitation of Hegar's sign if the fibroid is in front of the uterus. If the patient has menstruated profusely or suffers from irregular hæmorrhage—this often occurs in pregnancy with a dead fœtus—it is well in such cases to wait four weeks. If the case is one of pregnancy and the fœtus is dead the uterus will have become smaller and firmer and contracted upon the fœtus—a most important sign of fœtal death, whereas in the case of fibroid tumour the uterus will remain the same size. In the case of a living fœtus being present there would be enlargement. Distended bladder should not be mistaken for pregnancy, though it sometimes is sufficiently puzzling. Hæmometra—a collection of blood in the uterine cavity—is sometimes mistaken, but in this case there is a history of a long period of amenorrhœa which should raise a suspicion that the case is not one of pregnancy. The enlargement of the uterus is globular, the tumour tense and fluid—not doughy, no fœtal heart-sounds and no uterine souffle are heard. A vaginal septum or an imperforate hymen may be discovered.

